# Magnetogenetic stimulation inside MRI induces spontaneous and evoked changes in neural circuits activity in rats

**DOI:** 10.3389/fnins.2024.1459120

**Published:** 2024-10-01

**Authors:** Kai-Hsiang Chuang, Chunqi Qian, Assaf A. Gilad, Galit Pelled

**Affiliations:** ^1^School of Biomedical Sciences, The University of Queensland, Brisbane, QLD, Australia; ^2^Australian Research Council Training Centre for Innovation in Biomedical Imaging Technology, Brisbane, QLD, Australia; ^3^Department of Radiology, Michigan State University, East Lansing, MI, United States; ^4^Department of Chemical Engineering and Materials Science, Michigan State University, East Lansing, MI, United States; ^5^Department of Mechanical Engineering, Michigan State University, East Lansing, MI, United States

**Keywords:** magnetogenetics, connectivity, neuromodulation, neurostimulation, visual cortex

## Abstract

The ability to modulate specific neural circuits and simultaneously visualize and measure brain activity with MRI would greatly impact our understanding of brain function in health and disease. The combination of neurostimulation methods and functional MRI in animal models have already shown promise in elucidating fundamental mechanisms associated with brain activity. We developed an innovative magnetogenetics neurostimulation technology that can trigger neural activity through magnetic fields. Similar to other genetic-based neuromodulation methods, magnetogenetics offers cell-, area-, and temporal-specific control of neural activity. The magnetogenetic protein—Electromagnetic Perceptive Gene (EPG)—is activated by non-invasive magnetic fields, providing a unique way to target neural circuits by the MRI static and gradient fields while simultaneously measuring their effect on brain activity. EPG was expressed in rat's visual cortex and the amplitude of low-frequency fluctuation, resting-state functional connectivity (FC), and sensory activation was measured using a 7T MRI. The results demonstrate that EPG-expressing rats had significantly higher signal fluctuations in the visual areas and stronger FC in sensory areas consistent with known anatomical visuosensory and visuomotor connections. This new technology complements the existing neurostimulation toolbox and provides a means to study brain function in a minimally-invasive way which was not possible previously.

## Introduction

The neural circuit of the brain underpins our behavior and cognition. The capability of modulating a targeted circuit lies great potential for improving cognitive performance or relieving disease symptoms. The expansion of molecular and biological tools to modulate neural function can lead to improved and novel approaches to deliver neuromodulation that can offer cell-precise, temporally precise, region-specific, and non-invasive way to manipulate cellular function. The ability to modulate specific neural circuits and simultaneously visualize and measure brain activity would greatly impact the understanding of brain function in health and disease. MRI is a powerful tool for mapping brain-wide neural activity and connectivity induced by targeted neural activation. Developing technologies that will allow to trigger or suppress neural activity of a specific population of cells, while simultaneously acquiring neural activity via MRI methods remains an active area of research. Over the past decade, the combination of MRI with other neurostimulation methods such as implantable electrodes (Zhang et al., 2020), optogenetics (Li et al., 2011, 2014; Lee et al., 2010; Duffy et al., 2020; Chuang et al., 2023), chemogenetics (Li et al., 2023), and transcranial magnetic stimulation (Zhong et al., 2021) have elucidated several fundamental neural mechanisms including hemodynamic signals, neuroplasticity, resting-state fMRI, memory consolidation, and epilepsy.

A new neuromodulation method that can trigger neural activity through magnetic fields would provide an innovative and accessible way to manipulate neural circuits *in vivo*. Magnetogenetic*s* is the field of manipulating cells by magnetoreception-inspired bioengineering of novel genetic tools. For example, radio-waves and magnetic fields used for heating iron nanoparticles conjugated to ion channels were used to remote control cellular activity (Huang et al., 2010; Chen et al., 2015). Other studies reported on engineering two artificial chimeric magneto-sensors where ion channels were fused to ferritin (Stanley et al., 2015; Wheeler et al., 2016). Nevertheless, the ability to sense and respond to magnetic fields is well documented in many species, especially in diverse groups of fishes (Naisbett-Jones and Lohmann, 2022). Recently, we discovered a novel *electromagnetic perceptive gene* (EPG) from the glass catfish *Kryptopterus vitreolus* (Krishnan et al., 2018; Hunt et al., 2021). Recent work demonstrated that EPG, a 13.3 kD protein, is anchored via glycosylphosphatidylinositol (GPI) anchor to the cell membrane facing the extracellular space and increased intracellular calcium upon activation with electromagnetic field (EMF) (Ricker et al., 2023). EPG may undergo conformational changes when exposed to an EMF (Grady et al., 2022). Calcium imaging in cell culture (Krishnan et al., 2018) and electrophysiology recordings in rat brain slices (Metto et al., 2023) have demonstrated that EMF stimulation of EPG-expressing neurons leads to a significant response. In these experiments, an electromagnet device delivering 50–150 milli-Tesla (mT) was positioned over the cell culture and the brain slice to deliver the magnetic stimulation (Ashbaugh et al., 2021). Furthermore, it was demonstrated that this technology can induce behavioral changes at a level comparable to more conventional neurostimulation methods. For example, in a rat model of peripheral nerve injury, rats that expressed EPG in excitatory neurons in the somatosensory cortex showed improved sensorimotor performance when they were subjected to a magnetic stimulation (Cywiak et al., 2020). In a rat model of temporal lobe epilepsy, rats that expressed EPG in inhibitory interneurons in the hippocampus showed less seizures when injected with kainic acid (Metto et al., 2023). Thus, magnetogenetics could provide cell-, area- and temporal-specific features that a neuromodulation strategy requires, with the advantage of minimal invasiveness and technical challenges associated with electrophysiology or illumination inside an MRI scanner.

The EPG has been shown to be active in response to local magnetic fields in the order of mT. Nevertheless, it remains unclear how EPG activation may affect the activity and functional connectivity of specific brain circuits, and what is the range of magnetic fields that activates the EPG. The former is essential for understanding how EPG activation alters brain function and associated behavior. The latter would be crucial to continue and artificially design and synthesize new EPGs that could be sensitive and tunable to different magnetic field strengths and thus useful for different biological and physiological applications. The goal of this study was to understand whether functional imaging, such as fMRI, may be used to elucidate brain-wide modulation by EPG.

The anatomical projections and functional connectivity of the rat's visual cortex are well documented (Oh et al., 2014; Miller and Vogt, 1984; Nasrallah et al., 2016; Pelled and Goelman, 2004) and neuromodulation of this area also could have translational implications (Farnum et al., 2018; Farnum and Pelled, 2020). In this study, we evaluated how EPG expression in the rodent's visual cortex would influence the amplitude of low-frequency fluctuation, resting-state functional connectivity (FC), and sensory activation using a 7T MRI. We hypothesized that the magnetic fields of MRI can trigger EPG which will increase neural firing rates in these neurons and leading to activity changes in the interconnected regions. Therefore, in this proof-of-concept study, we expected (1) increased spontaneous activity at the EPG-injection site, (2) functional connectivity changes in the visual pathway and its connected areas, and (3) responsiveness changes to visual stimulation. We found that EPG activation by MRI increased fluctuation amplitude and connectivity between cortical and subcortical areas. Furthermore, visual evoked responses in EPG-expressing rats were larger compared to controls. Thus, magnetogenetics will allow inducing or silencing neural activity within an MRI. It would open a new array of possibilities to study brain circuits in health and disease.

## Methods

### Animal preparation

All procedures were approved by the Institutional Animal Care and Use Committee at Michigan State University. Adult male and female Long Evans rats (200–400 g, *n* = 10, 6 females, 4 males) were anesthetized with isoflurane (5% for induction; 2.5% for maintenance and surgery) and positioned in a stereotaxic frame. Stereotaxic injections of 5 μl at each location contained AAV1-CaMKIIα -{EPG(Rat)X3Flag}:IRES:EGFP at a titer of 10^13^ GC/ml (*n* = 5). This viral vector contained the gene encoding to EPG and a reporter gene, the Green Fluorescent Protein (GFP). Sham-control rats were injected with AAV-CaMKIIα-EGFP (*n* = 5). This viral vector contained only the reporter gene, GFP. The GFP only exhibits green fluorescence, and does not have any impact on neural activity. See Supplementary material for DNA sequence detail. The EPG sequence can be found in the gene bank: (GenBank: MH590650.1). The microinjector was positioned at 4 locations in the visual cortex: AP −7.3 mm, ML 3.2 mm; AP −7.3 mm, ML −3.2 mm; AP −5.4 mm, ML 4 mm; and AP −5.4 mm, ML −4 mm. Two to four weeks after stereotaxic injections MRI measurements took place. The experimental, EPG group, consisted of 5 rats: 2 males and 3 females, with an average weight of 380.6 ± 177.9 g, and the control, GFP group, also consisted of 5 rats: 2 males and 3 females, with an average weight of 338.2 ± 74.9 g. Student *T*-test showed that there was no difference between groups' weight (*p* = 0.63). For MRI the anesthesia was induced with 5% isoflurane, followed by a bolus injection of dexmedetomidine (0.05 mg/kg, sc; Dexdomitor^®^, Orion Pharma), after which isoflurane was discontinued, and a constant dexmedetomidine infusion was administered subcutaneously based on animal's weight (0.1 mg/kg/hr). During fMRI measurements, rats' temperature was maintained at 37°C, and the breathing rate, partial oxygen saturation, and heart rate were continuously monitored.

### MRI data acquisition

Data acquisition was performed using a 7T MRI system (Bruker BioSpec, Germany) with a volume coil for transmission and a 4-channel brain array (Bruker T11483V3) for reception. High-resolution anatomical scans were acquired using T2-weighted fast spin echo with a spatial resolution of 100 × 100 × 650 μm and with TR = 4.2 s, TE = 24 ms, RARE factor = 4, Slice Number = 32. Functional scans were acquired using single-shot gradient-echo echo planar imaging with TR = 1,000 ms, TE = 20 ms 40 × 40 matrix and 32 slices with a spatial resolution of 650 × 650 × 650 μm. Resting state fMRI scans were acquired over 12 min (720 scans). Afterwards, two kinds of sensory stimulations were used. The first was forepaw stimulation for verifying that the functional response is in the appropriate location, such as primary somatosensory cortex forelimb area (S1FL). The second was visual stimulation to evaluate the influence of the visual EPG on the visual activation. The electrical forepaw stimulation consisted of a 3 Hz pulse train of 0.5 mA applied to the right forepaw. Visual stimulation was delivered to both eyes using fiber optic cables presenting 5 Hz flashing lights. Both kinds of stimulations had alternating ON/OFF block design starting with 30-second OFF, then alternating 20-second ON and 20-second OFF for two times, leading to 110 total scans.

### MRI analysis

The MRI data were processed using MATLAB (MathWorks Inc), FSL (version 6.0, https://www.fmrib.ox.ac.uk/fsl), AFNI (version 18, National Institutes of Health, USA), and ANTs (v2.3.1, http://stnava.github.io/ANTs). The fMRI preprocessing followed an optimized pipeline we developed (Chuang et al., 2019). The first 5 scans were discarded to ensure that the baseline signal reached steady state. Potential motion artifact was corrected by FSL mcflirt, followed by brain extraction automatically using PCNN3D (Chou et al., 2011) and manual editing. Nuisance signals, including quadratic drift, 6 motion parameters and their derivatives, 10 principal components from tissues outside the brain which included muscle and scalp were extracted (Chuang et al., 2019). The time-series intensity of each voxel was normalized by the mean signal of the first OFF period (30 s) to convert the BOLD signal into percent signal change. The data was coregistered to the 0.3-mm SIGMA rat brain template (Barrière et al., 2019) via the structural T2-weighted MRI by linear and non-linear transformations using ANTs. The data were then smoothed by a 1.0 mm 3D Gaussian kernel. The stimulus-evoked fMRI data were high-pass filtered at 0.01 Hz to account for any potential baseline fluctuation. A general linear model was used to detect the evoked activation by convoluting the stimulus paradigm with a rodent hemodynamic response function (Lambers et al., 2020) and included the nuisance signals as confound regressors in the design matrix. The fitted coefficient beta was regarded as the activation level in voxel-wise and ROI-wise statistics.

For resting-state functional connectivity, the nuisance signals were first regressed out and the data were band-pass filtered at 0.01–0.1 Hz. To avoid the filter artifact, the first and last 15 time points were removed. Seed-based correlation analysis was used to measure FC across the brain. Based on the SIGMA template, the brain was divided into 170 bilateral gray matter regions-of-interest (ROIs) over the whole brain (see Supplementary Table S1 for a complete list of brain regions and abbreviations). The averaged time-course of each brain region was extracted as a seed signal. Pearson's correlation coefficients between time-courses in each voxel and ROI were calculated using AFNI 3dNetCorr. Fisher's *z*-transformation was used to convert correlation coefficients to *z* values. Furthermore, the amplitude of low frequency fluctuation (ALFF) was used to measure the spontaneous activity change induced by EPG. The spectral power of voxel time-course with nuisance removal but without band-pass filter was calculated. The total power within the 0.015 to 0.2 Hz range was used as the ALFF. The fractional ALFF (fALFF) was calculated by normalizing the ALFF by the total power above 0.015 Hz.

### Immunohistochemistry

Rats were perfused transcardially with 1X Phosphate Buffered Saline (PBS) and 4% Paraformaldehyde (PFA). The brains were extracted and placed in 4% PFA overnight after which they were placed in varying concentrations of sucrose. Frozen brains were sectioned at a thickness of 50 μm and slices were placed in 4C in PBS. Slices were washed in PBST and a blocking solution added (donkey serum, Sigma-Aldrich, St. Louis, MO). Slices were then incubated overnight with the primary antibodies (1:500, Anti-CamKII, ab52476, and Anti-GFP, ab13970, Abcam, Boston, MA). The following day, slices were washed and incubated with the secondary antibodies (1:500, Donkey Anti-Rabbit conjugated with Alexa Fluor 657, and Donkey Anti-Chicken conjugated with Alexa Fluor 488, Jackson ImmunoResearch Labs, West Grove, PA). Images were acquired using the Biotek Cytation 5 Cell Imaging Multi-Mode Reader (Biotek Instruments, Inc., Winooski, VT) configured on an inverted microscope.

### Statistical analysis

Within and between group comparison was conducted in each voxel or ROI. Voxel-wise group comparison was calculated by one-sample *t-*test and thresholded at *p* < 0.01 (False Discovery Rate [FDR] corrected). Between-group differences were calculated by two-sample *t-*test and thresholded at *p* < 0.05 (FDR corrected). For the evoked fMRI, the beta maps were compared, and for the resting-state FC the z-score maps were compared. Voxel-wise *t*-test was conducted using AFNI 3dttest++ with the t-map output converted to z-score map using the “-toz” option. The resting-state fALFF of each ROI was compared using 2-way ANOVA (Prism 9, GraphPad Software). For visualization, significant connections were overlaid on the 3D-rendered brain atlas using BrainNet Viewer (https://www.nitrc.org/projects/bnv/).

## Results

**Figure 1:**
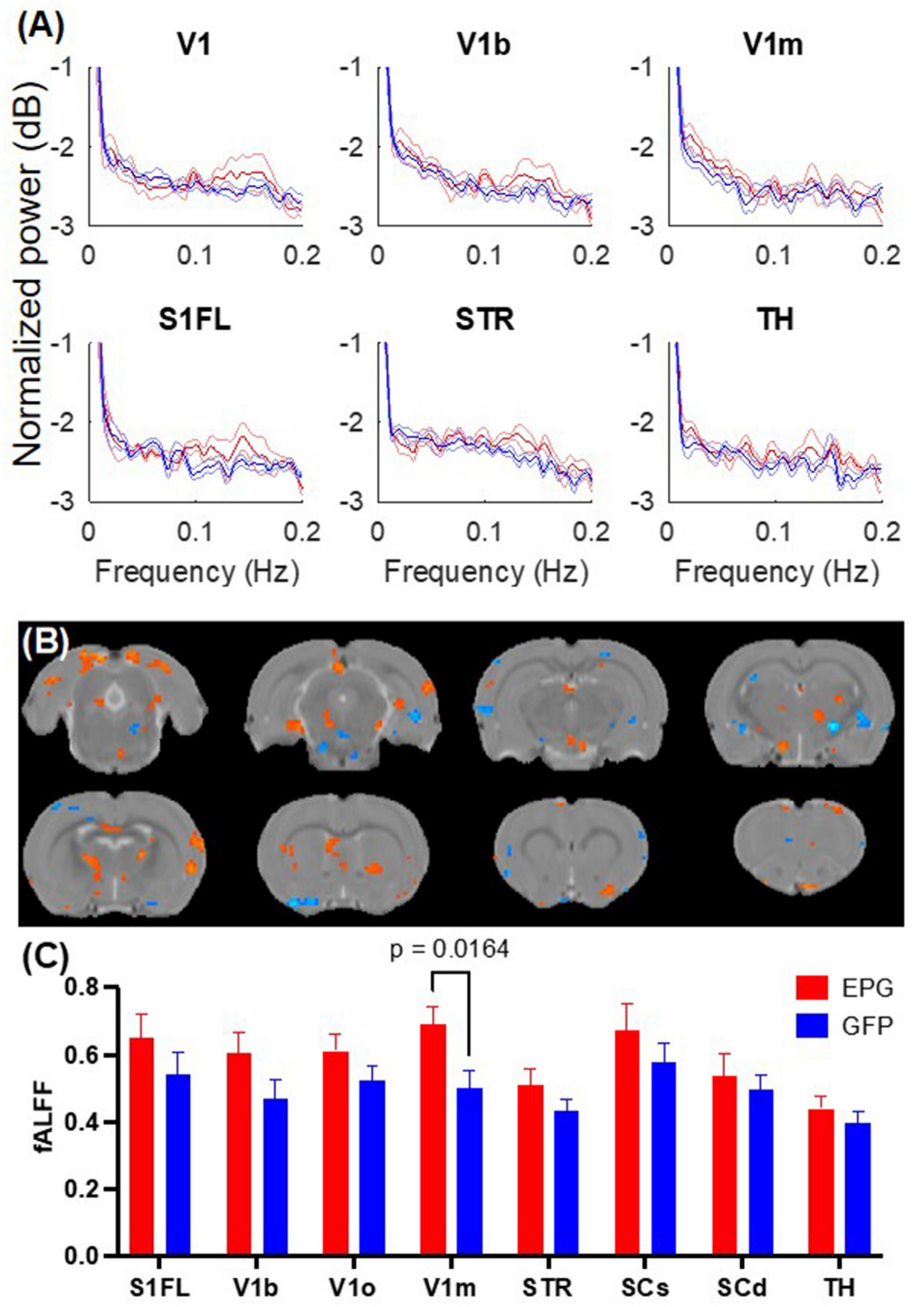
EPG expression in the visual cortex leads to increases in fractional amplitude of low frequency fluctuation (fALFF). **(A)** Power spectra of the resting-state fMRI signals from selected brain regions show elevated signal fluctuations above 0.1 Hz in the EPG (red) compared to the GFP (blue) group. The solid line represents the mean and the shaded error bar represents the standard error of mean (SEM). **(B)** Voxel-wise comparison of fALFF between the EPG and GFP groups (two-sample *t-*test, *p* < 0.05, FDR corrected). **(C)** Regional analysis of the fALFF demonstrated that EPG rats exhibit significantly larger fluctuations at the primary visual cortex (V1m) compared to control rats [Two-way ANOVA, *F*_(1, 64)_ = 12.88, *p* = 0.0006]. This increased spontaneous neural activity in the region that the EPG was expressed suggests that EPG could be activated by the magnetic field of the MRI. See Supplementary Table S1 for abbreviations of brain regions.

Resting-state fMRI images were collected immediately after positioning the rat in the scanner to determine whether the magnetic fields of MRI would activate the EPG and alter the spontaneous neural activity. The signal fluctuation amplitude and spectral power at the resting state were measured for 12 min. Unlike a typical resting-state fMRI study that only inspects fluctuations below 0.1 Hz, we observed larger changes between 0.1 to 0.2 Hz (Figure 1A). Based on the spectral analysis, we calculated the fALFF within the 0.015 to 0.2 Hz range. Voxel-wise comparisons show sparse areas with significantly higher signal fluctuation amplitudes such as the retrosplenial cortex (RSC) (Figure 1B). Regional analysis shows a significantly larger fALFF in the EPG group [Two-way ANOVA, *F*_(1, 64)_ = 12.88, *p* = 0.0006] with the most significant change found in the V1m (*p* = 0.016, Fisher's LSD test) (Figure 1C).

**Figure 2:**
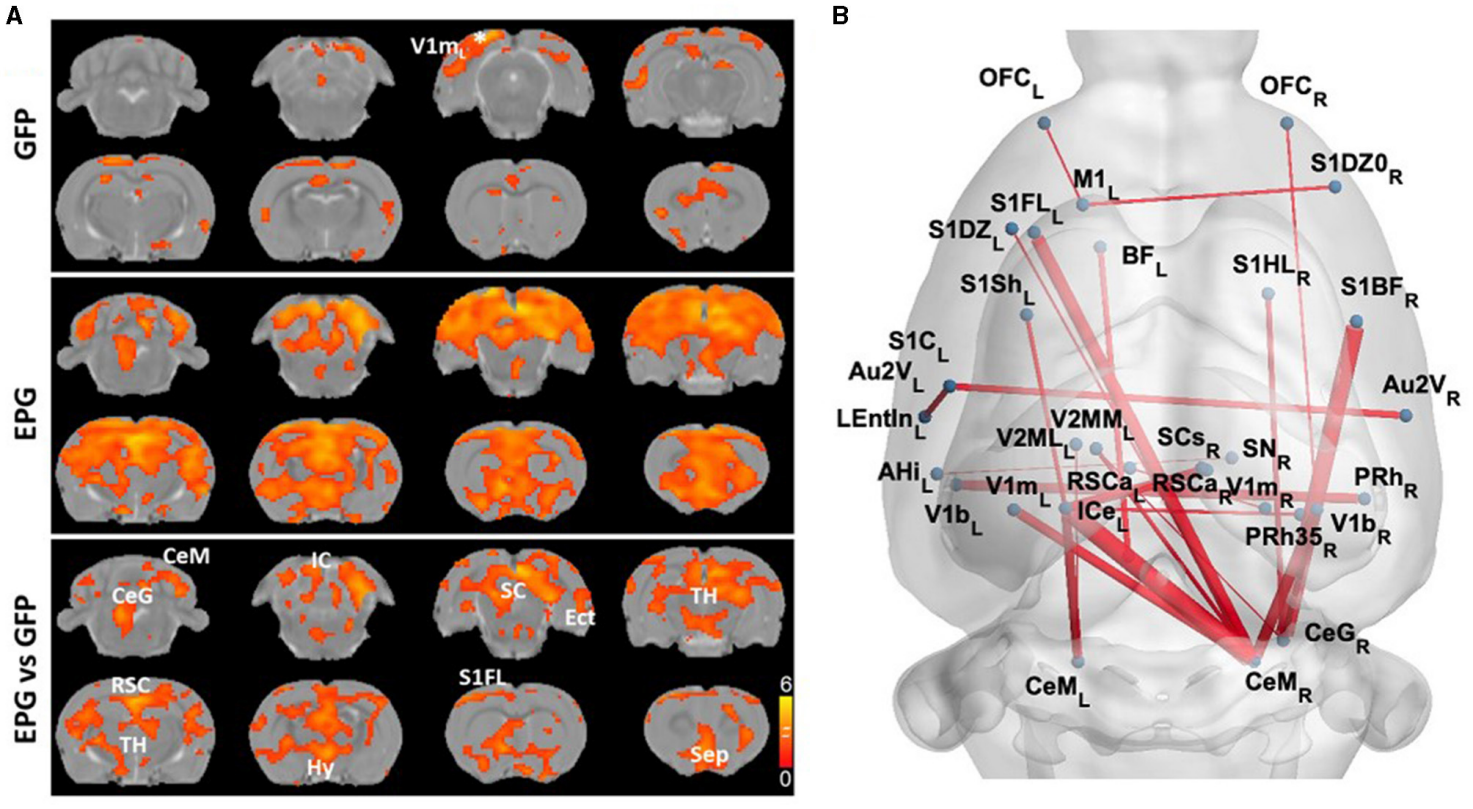
EPG expression in the visual cortex leads to increases in Functional Connectivity (FC) across cortical and subcortical areas. **(A)** A seed ROI was placed in the V1m of the left hemisphere, where the EPG was expressed. In the GFP control group, FC was mostly in the visual cortex with certain connectivity with the medial prefrontal cortex (one-sample *t*-test, *p* < 0.01 FDR corrected). In the EPG group, broadly increased FC can be seen over the whole brain. Between group comparison shows significantly higher connectivity between brain areas of EPG rats compared to controls (two-sample *t*-test, *p* < 0.05 FDR corrected). **(B)** Whole brain FC (two-sample *t*-test, *p* < 0.001 uncorrected) shows extensively increased connectivity beyond the visual cortex. See Supplementary Table S1 for abbreviations of brain regions.

To further understand the effects of EPG activation on distant brain regions, we calculated the inter-regional signal correlation as a measure of FC in EPG-expressing rats. With a seed ROI at the V1m, broadly distributed FC can be seen throughout the cortex and subcortical areas, such as the RSC, S1, SC, TH, hypothalamus (Hy), and septum (Sep) (Figure 2A, *p* < 0.05 FDR corrected). Whole brain analysis revealed increased FC between the cortical areas, including the primary and secondary visual, retrosplenial, entorhinal, perirhinal, primary somatosensory, auditory, motor, and orbitofrontal cortices, as well as with some subcortical areas, such as the basal forebrain (BF), SC and IC, and the cerebellum (Figure 2B, *p* < 0.001 uncorrected).

**Figure 3:**
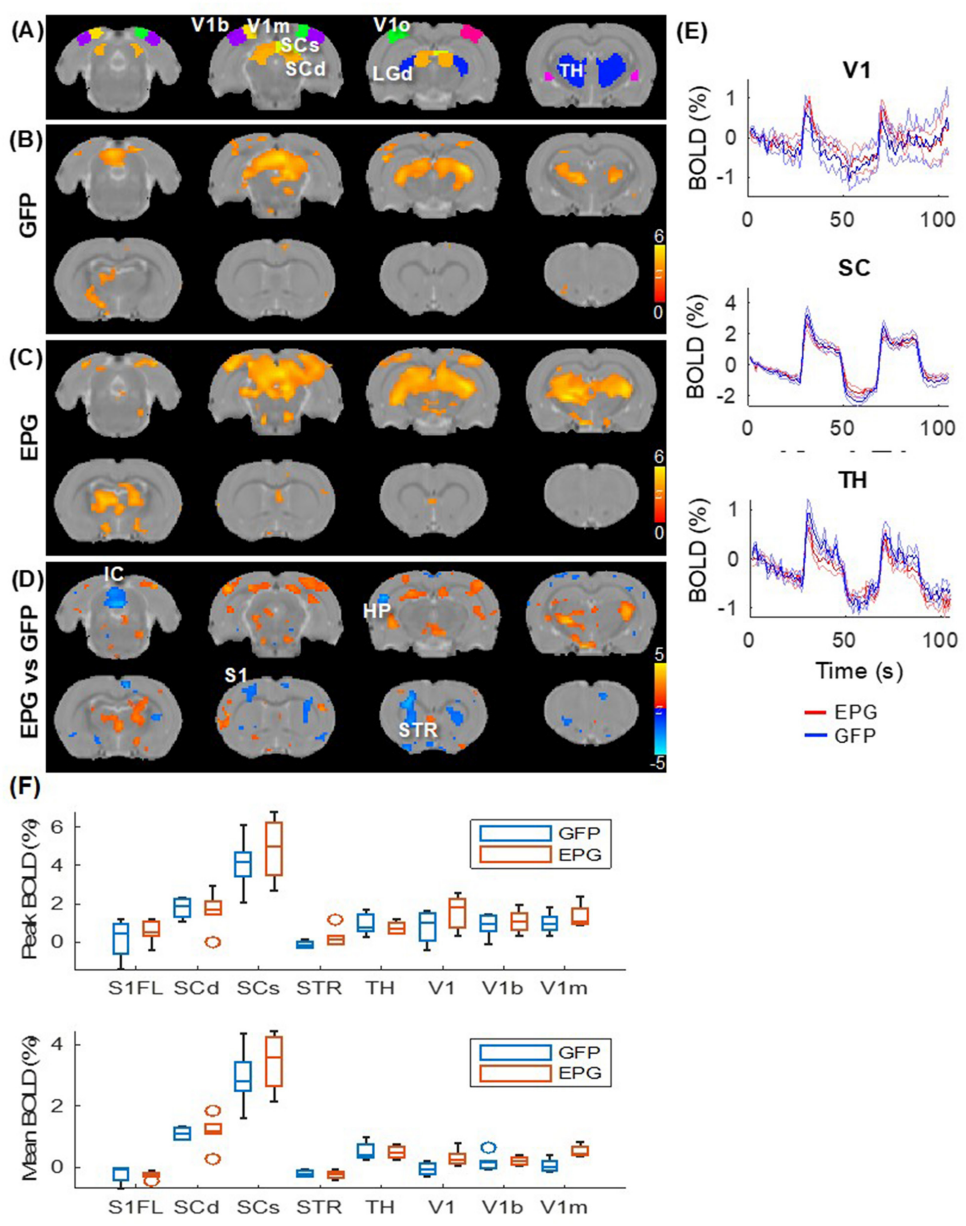
Evoked visual responses. **(A)** ROI in the visual pathway and the thalamus, with the LGd of the thalamus highlighted in the 3^rd^ slice. Two epochs of 20s visual stimulation were delivered, leading to activation in the visual pathway in the **(B)** GFP and **(C)** EPG groups (one-sample *t-*test, *p* < 0.01 FDR corrected). **(D)** The EPG group showed significant increased activation in the primary visual cortex (V1), hippocampus (HP) and ventral thalamus, and decreased activation in the striatum (STR) and the inferior colliculus (IC), compared to control rats (two-sample *t*-test, *p* < 0.05, FDR corrected). **(E)** Averaged signal time-courses from selected ROI. The solid line represents the mean, the shaded error bar represents SEM and the gray bars indicate the stimulation periods. **(F)** The peak and averaged BOLD signal changes during the activated periods.

Under visual stimulation, rats expressing the control virus (GFP group; Figure 3B) exhibit focal activation (*p* < 0.01, FDR corrected) in the visual pathway, including the dorsal lateral geniculate nucleus of the thalamus (LGd), superior colliculus superficial/deep sub-regions (SCs/SCd) to the primary visual cortex (V1) and its binocular (V1b) and monocular (V1m) sub-regions. Rats in the EPG group (Figure 3C) show not only stronger activation in the visual pathway but also more distributed activation in the entire thalamus (TH). Comparing the EPG with the control group (*p* < 0.05, FDR corrected; Figure 3D), stronger activation was seen in the V1, hippocampus (HP), and ventral thalamus. A decreased signal was seen in the striatum (STR), inferior colliculus (IC), and cerebellum. Interestingly, reduced activation is also seen in the primary somatosensory cortex (S1). Comparing the averaged BOLD signal time-courses in the V1, SC, and TH, the responses were comparable between the EPG and GFP-control groups (Figure 3E), indicating that the EPG did not alter the hemodynamic response function. Unlike the subcortical areas, the BOLD activation in the V1 shows a faster decrease, consistent with previous studies (Niranjan et al., 2016). A slightly higher and less variable activation with a much smaller standard deviation among individuals was seen in EPG group. Overall, EPG modulated the visual activation in distributed areas throughout the brain, though the activation amplitude and shape did not change much.

**Figure 4:**
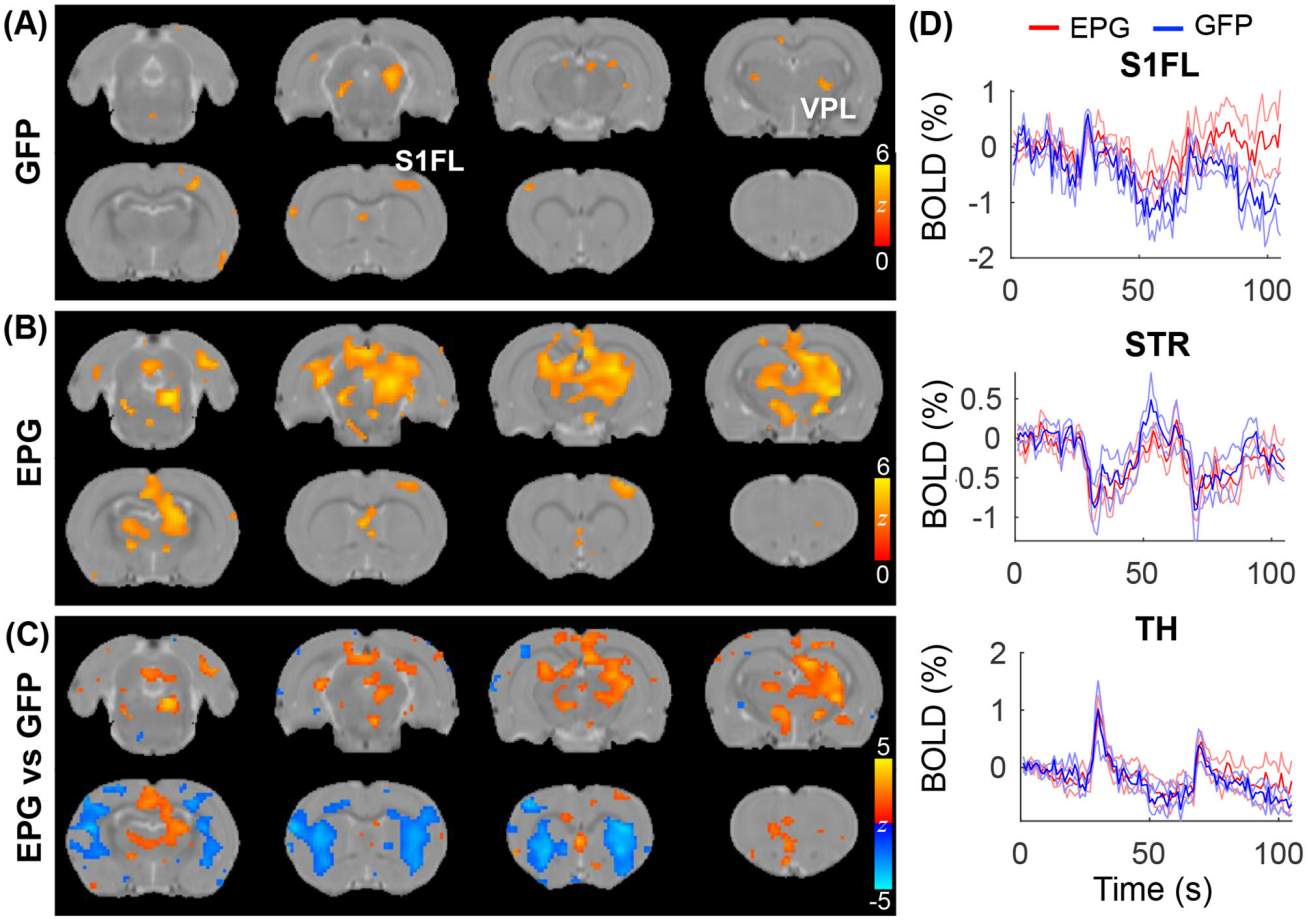
Evoked tactile responses. Two epochs of 20s tactile stimulation were delivered to the forepaw of the rat, leading to significant activation in the somatosensory pathway in the **(A)** GFP and **(B)** EPG groups (one-sample *t-*test, *p* < 0.01 FDR corrected). **(C)** Between group comparison shows that the EPG group had increased activation in the Thalamus (TH) and in the anterior cingulate cortex, and decreased activation in the striatum (STR), compared to control rats (two-sample *t-*test, *p* < 0.05, FDR corrected). **(D)** Averaged signal time-courses from selected ROI. The solid line represents the mean, the shaded error bar represents SEM and the gray bars indicate the stimulation periods.

With the broad cortical and subcortical changes in FC (Figure 2) and under visual stimulation (Figure 3), we hypothesized that visual cortical EPG activation can modulate other sensory pathway, such as the somatosensory forelimb, activity. To test the effects of visual EPG on the somatosensory pathway, we conducted electrical forepaw stimulation. As expected, activation in the ventral posterior lateral (VPL) thalamus, and primary somatosensory forelimb area (S1FL) were detected in the GFP-control group (Figure 4A; *p* < 0.01 FDR corrected). Interestingly, whereas the activation in the S1FL was comparable, much broader activation in the thalamus, anterior cingulate cortex was seen in the EPG group (Figure 4B). Comparison between the EPG and control groups revealed increased activation in the TH but decreased activation in the STR (Figures 4C, D), similar to that under visual stimulation.

**Figure 5:**
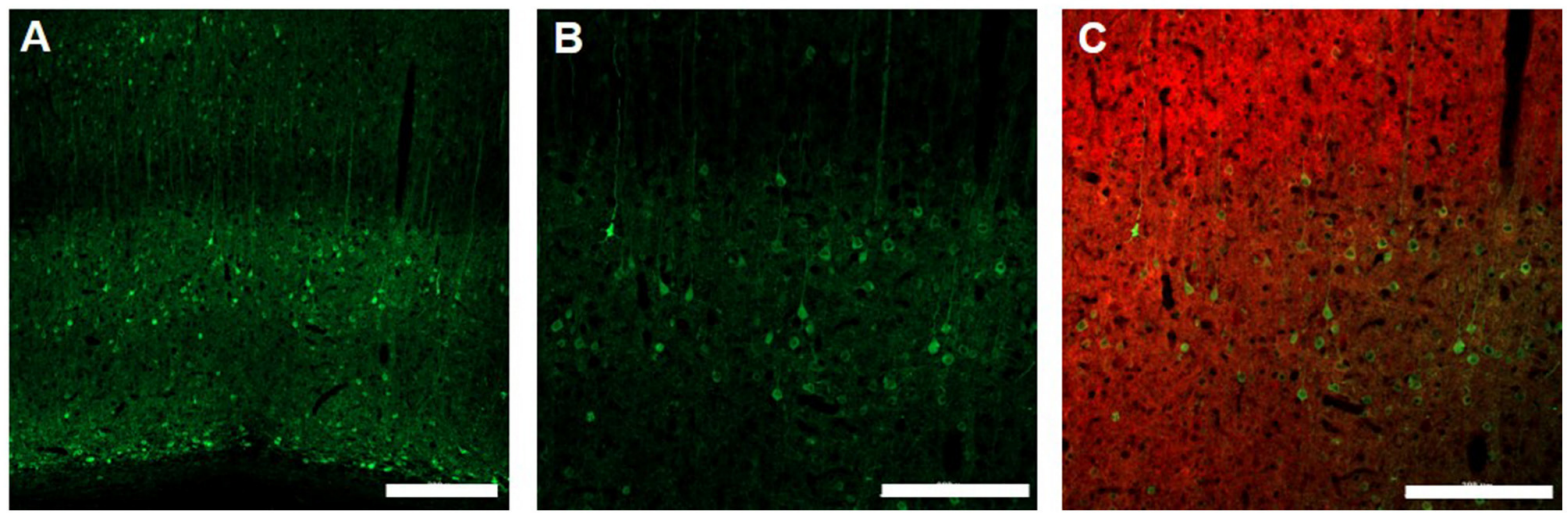
Immunohistology of EPG expression in the visual cortex. 10X magnification **(A)**, Scale bar = 300 μm] and 20X magnification **(B)**, Scale bar = 200 μm of EPG (labeled with green, anti-GFP) throughout the layers of the visual cortex in excitatory neurons (labeled with red, anti-CamKII) **(C)**, Scale bar = 200 μm.

Finally, at the end of the fMRI experiments rats were sacrificed for immunohistology validation of EPG expression. Figure 5 shows the expression of EPG (green fluorescence) in excitatory neurons (red fluorescence) in the V1m, indicating wide distribution across the cortical layers.

## Discussion

Previous work has shown that electromagnetic devices positioned over EPG-expressing neurons induce changes in neural activity *in vivo* and *in vitro*. Here, we demonstrated that EPG can also be activated by strong magnetic fields induced by an MRI, including both the static and the oscillating gradient fields. FC analysis demonstrated that EPG-expressing rats showed extensively stronger FC in sensory areas, and fALFF analysis demonstrated higher signal fluctuation, specifically in the visual areas. These results suggest that by using EPG-based magnetogenetics, MRI can induce changes in the connectivity and activity of neural circuits in rodents. This new approach would allow studying the activity of specific circuits associated with different brain function and disease conditions in a way that was not possible previously.

The molecular structure of the EPG and how it responds to magnetic fields is an active area of research. Strong evidence suggests that exposure to magnetic fields leads to conformational changes in the EPG protein. By fusing split fragments of a certain protein to both termini of the EPG, the fragments can be reassembled into a functional protein under magnetic stimulation due to a conformational change (Grady et al., 2022). Electrophysiology recordings in acute brain slices obtained from rats that expressed EPG in the somatosensory cortex show that the time course of neural responses induced by EPG activity is in the order of milliseconds; Magnetic stimulation significantly increased action potentials within 100 ms, and the number of action potentials returned to baseline afterward (Metto et al., 2023). Thus, the molecular dynamics of the EPG allow a rapid and controlled activation of neural activity. Indeed, the results from the current fMRI support this hypothesis.

It is essential to determine the precise magnetic field strength required to activate EPG. Studies have consistently demonstrated that exposure to magnetic fields in the mT range can activate a variety of cells and neurons via EPG (Krishnan et al., 2018; Grady et al., 2022; Ashbaugh et al., 2021; Cywiak et al., 2020; Hwang et al., 2020). Nevertheless, no test has been carried out with a stronger magnetic field to date. The stimulation frequency response of EPG is also unclear. Indeed, most active proteins are in unstable thermodynamic states and, therefore may not require much energy for the activation (Jones, 2016). For example, bacterial enzyme kinetics are affected by low magnetic fields via spin-orbit coupling when bound to Xenon gas (Anderson et al., 2001). Moreover, small magnetic fields (~3 mT) can affect the enzymatic reaction of horseradish peroxidase via a similar mechanism (Chalkias et al., 2008). These reports indicate that magnetic fields can directly affect proteins by mechanisms other than the movement or alignment of a magnetic core. Furthermore, there is mounting evidence that biochemical reactions can significantly amplify the magnetic field effect (Kattnig et al., 2016) and that chemical inhibitors can reverse such effect (Marley et al., 2014). Although MRI appears to have a magnetic field strong enough to activate EPG and alter neuronal activity, additional research is required to determine the extent to which the permanent magnetic field of the MRI and the lower, fluctuating magnetic field generated by the EPI gradients contribute to this effect.

In the current study, we started collecting resting-state fMRI because it is possible that just exposing the EPG-expressing rodent to the strong magnetic field of the MRI scanner would activate the EPG. After acquiring 12 min of resting-state fMRI we collected evoked response fMRI. Changes in spontaneous neural activity, connectivity, and sensory evoked responses were observed in fALFF, FC, and fMRI analysis. The activity and connectivity at many remote regions from the EPG-expressing site was observed. Based on the axonal connectivity mapping (Oh et al., 2014; Miller and Vogt, 1984), V1 has extensively direct projections to the ACC, RSC, SC, S1, motor cortex and auditory cortex to support visuosensory and visuomotor integration in visually guided behaviors. Therefore, the observed change is mostly likely due to the EPG modulation in these downstream pathways. Consistent with the structural and functional connectivity findings, we found somatosensory activation under a tactile stimulation can be modulated by EPG likely via the visuosensory connections. Additionally, the visual and somatosensory cortices directly project to the dorsal striatum to support the sensorimotor functions and action selection (McGeorge and Faull, 1989; Reig and Silberberg, 2014). This is consistent with our observation of altered striatal activation under the visual or tactile stimulation though its functional role remains to be elucidated. In future experiments it will be interesting to use additional tools to colocalize the MRI findings with specific cell population and location. We and others have shown that immunohistochemistry analysis of immediate early genes are associated with increased fMRI responses (Zhong et al., 2021; Verley et al., 2018). Future studies could continue and reveal the local and downstream pathways associated with magnetogenetics modulation. Furthermore, recent reports indicate that body weight, age and sex may be related to brain volume and functional connectivity (Labounek et al., 2024; Raitamaa et al., 2024). In the future, and with larger cohorts, it will be important to investigate if change in resting-state fMRI and functional connectivity induced by EPG is affected by these factors as well.

Besides detecting the effects induced by the MRI magnetic fields, an intriguing question is whether one could image activity induced by an electromagnetic device, such as transcranial magnetic stimulation (TMS). As any electromagnetic device can suffer from MRI interference and affect MRI quality, previous studies have developed compatible hardware and interleave acquisition protocols for concurrent TMS-fMRI experiments (Riddle et al., 2022). Therefore, similar approach could be adapted to activate EPG inside MRI. To minimize the effects induced by the oscillating gradient fields of the EPI scan, one may position the EPG-expressing brain region at the isocenter, where the gradient remains 0. However, the effects induced by the static magnetic field cannot be avoided. Alternatively, one may use other imaging modality, such as functional ultrasound or positron emission tomography, to map the brain-wide changes, or wide-field optical imaging to map changes in the exposed cortical surface.

The ability to facilitate or inhibit a population of neurons and visualize its effect on the entire brain and on specific brain circuits and connectivity in real-time has the potential to shed light on different disease mechanisms and therapeutic targets. For example, this approach could be useful to identify how positioning deep brain stimulation electrodes at different brain nuclei may affect directly and indirectly neural circuits in Parkinson's disease, other movement disorders, epilepsy, and depression. Combining whole-brain functional imaging and site-specific excitation and inhibition could also reveal the temporal and spatial sequences associated with sensory perception, learning and memory, and may identify new targets for interventions. Magnetogenetics would also be useful to target not only specific locations, but also specific cell types to study brain circuits (Choi et al., 2024). These approaches could also be applied to evaluate EPG-based treatments in disease models, such as using EPG in inhibitory neurons for suppressing epileptic seizure (Metto et al., 2023) or EPG for facilitating recovery from injury (Cywiak et al., 2020).

Previously, it was demonstrated that EPG can be expressed in different cell types by using specific neural promoters. For example, in a rat model of peripheral nerve injury, EPG was expressed in excitatory neurons in the primary somatosensory cortex under CamKII promoter (Cywiak et al., 2020) and in inhibitory interneurons in the hippocampus under hDlx promoter (Metto et al., 2023). Like optogenetics and chemogenetics such as designer receptors exclusively activated by designer drugs (DREADDS), the EPG can be delivered to any desired brain structure using molecular biology tools, and it can be expressed under cell-specific promoters. In addition, like optogenetics and electronic devices, the activation of cells expressing EPG is immediate and in the order of a 100 of milliseconds as was demonstrated in Metto et al. (2023). However, unlike optogenetics, chemogenetics, and electronic devices, activation of EPG is minimally invasive and does not require the administration of drugs or implantation of any device. Currently, it does require the delivery of viral vectors through stereotaxic injections into the brain. Nevertheless, new and upcoming approaches for minimally invasive gene delivery that are being designed for different health disorders are likely to offer new ways to deliver transgenes that can cross the blood-brain barrier and target a specific neural population (Smith et al., 2021; Prezelski et al., 2021; Samaranch et al., 2017) and will provide new approaches to deliver EPG into the brain. Furthermore, some evidence suggests that optogenetics often times evoke visual stimulation due to the unavoidable leakage of the excitation light, which creates problems for behavioral tasks involving visual processing. EPG can also achieve temporally precise control that DREADDS currently can't do. Thus, magnetogenetics technology eliminates many complications and side effects often associated with current stimulation techniques.

In summary, we demonstrated for the first time a new method to manipulate neural circuits in rodents via magnetic fields inside the MRI. With resting-state and evoked fMRI, we found EPG can modulate downstream pathways activity and FC extending from the visual cortex. This indicates a crucial need of using whole-brain functional imaging to understand downstream effects of a targeted neuromodulation. Further development of the magnetogenetics may provide a new tool for studying connectivity and neural activity in animal models.

## Data Availability

The datasets presented in this study can be found in online repositories. The names of the repository/repositories and accession number(s) can be found in the article/Supplementary material.

## References

[B1] Anderson M. A. Xu Y. Grissom C. B. (2001). Electron spin catalysis by xenon in an enzyme. J. Am. Chem. Soc. 123, 6720–6721. 10.1021/ja015949f 11439070

[B2] Ashbaugh R. C. Udpa L. Israeli R. R. Gilad A. A. (2021). Bioelectromagnetic platform for cell, tissue, and in vivo stimulation. Biosensors 11:248. 10.3390/bios11080248 34436050 PMC8392012

[B3] Barrière D. A. Magalhães R. Novais A. Marques P. Selingue E. Geffroy F.. (2019). The SIGMA rat brain templates and atlases for multimodal MRI data analysis and visualization. Nat. Commun. 10:5699. 10.1038/s41467-019-13575-7 31836716 PMC6911097

[B4] Chalkias N. G. Kahawong P. Giannelis E. P. (2008). Activity increase of horseradish peroxidase in the presence of magnetic particles. J. Am. Chem. Soc. 130, 2910–2911. 10.1021/ja7102263 18275197

[B5] Chen R. Romero G. Christiansen M. G. Mohr A. Anikeeva P. (2015). Wireless magnetothermal deep brain stimulation. Science 347, 1477–1480. 10.1126/science.1261821 25765068

[B6] Choi S. H. Shin J. Park C. Lee J. U. Lee J. Ambo Y.. (2024). In vivo magnetogenetics for cell-type-specific targeting and modulation of brain circuits. Nat. Nanotechnol. 19, 1333–1343. 10.1038/s41565-024-01694-2 38956320

[B7] Chou N. Wu J. Bai Bingren J. Qiu A. Chuang K. H. (2011). Robust automatic rodent brain extraction using 3-D pulse-coupled neural networks (PCNN). IEEE Trans. Image Process. 20, 2554–2564. 10.1109/TIP.2011.2126587 21411404

[B8] Chuang K. H. Lee H. L. Li Z. Chang W. T. Nasrallah F. A. Yeow L. Y.. (2019). Evaluation of nuisance removal for functional MRI of rodent brain. Neuroimage 188, 694–709. 10.1016/j.neuroimage.2018.12.048 30593905

[B9] Chuang K. H. Li Z. Huang H. H. Khorasani Gerdekoohi S. Athwal D. (2023). Hemodynamic transient and functional connectivity follow structural connectivity and cell type over the brain hierarchy. Proc. Natl. Acad. Sci. USA. 120:e2202435120. 10.1073/pnas.2202435120 36693103 PMC9945945

[B10] Cywiak C. Ashbaugh R. C. Metto A. C. Udpa L. Qian C. Gilad A. A.. (2020). Non-invasive neuromodulation using rTMS and the electromagnetic-perceptive gene (EPG) facilitates plasticity after nerve injury. Brain Stimul. 13, 1774–1783. 10.1016/j.brs.2020.10.006 33068795 PMC7722061

[B11] Duffy B. A. Choy M. Lee J. H. (2020). Predicting successful generation and inhibition of seizure-like afterdischarges and mapping their seizure networks using fMRI. Cell Rep. 30, 2540–2554.e2544. 10.1016/j.celrep.2020.01.095 32101734 PMC8720841

[B12] Farnum A. Li W. Udpa L. Shenoy B. B. Pelled G. (2018). “Designing an apparatus for behavioral testing in awake rodents during brain stimulation,” in 2018 11th Biomedical Engineering International Conference. 10.1109/BMEiCON.2018.860994125651158

[B13] Farnum A. Pelled G. (2020). New vision for visual prostheses. Front. Neurosci. 14:36. 10.3389/fnins.2020.00036 32132890 PMC7040096

[B14] Grady C. J. Schossau J. Ashbaugh R. C. Pelled G. Gilad A. A. (2022). A putative design for electromagnetic activation of split proteins for molecular and cellular manipulation. bioRxiv. 10.1101/2022.11.30.518522 38605993 PMC11007078

[B15] Huang H. Delikanli S. Zeng H. Ferkey D. M. Pralle A. (2010). Remote control of ion channels and neurons through magnetic-field heating of nanoparticles. Nat. Nanotechnol. 5, 602–606. 10.1038/nnano.2010.125 20581833

[B16] Hunt R. D. Ashbaugh R. C. Reimers M. Udpa L. De Jimenez G. S. Moore M.. (2021). Swimming direction of the glass catfish is responsive to magnetic stimulation. PLoS ONE 16:e0248141. 10.1371/journal.pone.0248141 33667278 PMC7935302

[B17] Hwang J. Choi Y. Lee K. Krishnan V. Pelled G. Gilad A. A.. (2020). Regulation of electromagnetic perceptive gene using ferromagnetic particles for the external control of calcium ion transport. Biomolecules 10:308. 10.3390/biom10020308 32075263 PMC7072303

[B18] Jones A. R. (2016). Magnetic field effects in proteins. Mol. Phys. 114, 1691–1702. 10.1080/00268976.2016.1149631

[B19] Kattnig D. R. Evans E. W. Déjean V. Dodson C. A. Wallace M. I. Mackenzie S. R.. (2016). Chemical amplification of magnetic field effects relevant to avian magnetoreception. Nat. Chem. 8, 384–391. 10.1038/nchem.2447 27001735

[B20] Krishnan V. Park S. A. Shin S. S. Alon L. Tressler C. M. Stokes W.. (2018). Wireless control of cellular function by activation of a novel protein responsive to electromagnetic fields. Sci. Rep. 8:8764. 10.1038/s41598-018-27087-9 29884813 PMC5993716

[B21] Labounek R. Bondy M. T. Paulson A. L. Bédard S. Abramovic M. Alonso-Ortiz E.. (2024). Body size interacts with the structure of the central nervous system: a multi-center in vivo neuroimaging study. bioRxiv. 10.1101/2024.04.29.591421 38746371 PMC11092490

[B22] Lambers H. Segeroth M. Albers F. Wachsmuth L. van Alst T. M. Faber C. A.. (2020). A cortical rat hemodynamic response function for improved detection of BOLD activation under common experimental conditions. Neuroimage 208:116446. 10.1016/j.neuroimage.2019.116446 31846759

[B23] Lee J. H. Durand R. Gradinaru V. Zhang F. Goshen I. Kim D. S.. (2010). Global and local fMRI signals driven by neurons defined optogenetically by type and wiring. Nature 465, 788–792. 10.1038/nature09108 20473285 PMC3177305

[B24] Li N. Downey J. E. Bar-Shir A. Gilad A. A. Walczak P. Kim H.. (2011). Optogenetic-guided cortical plasticity after nerve injury. Proc. Natl. Acad. Sci. USA 108, 8838–8843. 10.1073/pnas.1100815108 21555573 PMC3102379

[B25] Li N. van Zijl P. Thakor N. Pelled G. (2014). Study of the spatial correlation between neuronal activity and BOLD fMRI responses evoked by sensory and channelrhodopsin-2 stimulation in the rat somatosensory cortex. J. Molec. Neurosci. 53, 553–561. 10.1007/s12031-013-0221-3 24443233 PMC4104155

[B26] Li Z. Athwal D. Lee H.-. L Sah P. Opazo P. Chuang K- H.. (2023). Locating causal hubs of memory consolidation in spontaneous brain network in male mice. Nat. Commun. 14:5399. 10.1038/s41467-023-41024-z 37669938 PMC10480429

[B27] Marley R. Giachello C. N. Scrutton N. S. Baines R. A. Jones A. R. (2014). Cryptochrome-dependent magnetic field effect on seizure response in Drosophila larvae. Sci. Rep. 4:5799. 10.1038/srep05799 25052424 PMC4107376

[B28] McGeorge A. J. Faull R. L. (1989). The organization of the projection from the cerebral cortex to the striatum in the rat. Neuroscience 29, 503–537. 10.1016/0306-4522(89)90128-0 2472578

[B29] Metto A. C. Telgkamp P. McLane-Svoboda A. K. Gilad A. A. Pelled G. (2023). Closed-loop neurostimulation via expression of magnetogenetics-sensitive protein in inhibitory neurons leads to reduction of seizure activity in a rat model of epilepsy. Brain Res. 1820:148591. 10.1016/j.brainres.2023.148591 37748572

[B30] Miller M. W. Vogt B. A. (1984). Direct connections of rat visual cortex with sensory, motor, association cortices. J. Comp. Neurol. 226, 184–202. 10.1002/cne.902260204 6736299

[B31] Naisbett-Jones L. C. Lohmann K. J. (2022). Magnetoreception and magnetic navigation in fishes: a half century of discovery. J. Compar. Physiol. A 208, 19–40. 10.1007/s00359-021-01527-w 35031832

[B32] Nasrallah F. A. To X. V. Chen D. Y. Routtenberg A. Chuang K. H. (2016). Resting state functional connectivity data supports detection of cognition in the rodent brain. Data Brief 7, 1156–1164. 10.1016/j.dib.2016.03.041 27115031 PMC4833131

[B33] Niranjan A. Christie I. N. Solomon S. G. Wells J. A. Lythgoe M. F. (2016). fMRI mapping of the visual system in the mouse brain with interleaved snapshot GE-EPI. Neuroimage 139, 337–345. 10.1016/j.neuroimage.2016.06.015 27296012 PMC4988789

[B34] Oh S. W. Harris J. A. Ng L. Winslow B. Cain N. Mihalas S.. (2014). A mesoscale connectome of the mouse brain. Nature 508, 207–214. 10.1038/nature13186 24695228 PMC5102064

[B35] Pelled G. Goelman G. (2004). Different physiological MRI noise between cortical layers. Magn. Reson. Med. 52, 913–916. 10.1002/mrm.20229 15389942

[B36] Prezelski K. Keiser M. Stein J. M. Lucas T. H. Davidson B. Gonzalez-Alegre P.. (2021). Design and validation of a multi-point injection technology for MR-guided convection enhanced delivery in the brain. Front. Med. Technol. 3:725844. 10.3389/fmedt.2021.725844 35047955 PMC8757778

[B37] Raitamaa L. Kautto J. Tuunanen J. Helakari H. Huotari N. Järvel,ä M.. (2024). Association of body-mass index with physiological brain pulsations across adulthood - a fast fMRI study. Int. J. Obes. 48, 1011–1018. 10.1038/s41366-024-01515-5 38553569 PMC11216984

[B38] Reig R. Silberberg G. (2014). Multisensory integration in the mouse striatum. Neuron 83, 1200–1212. 10.1016/j.neuron.2014.07.033 25155959 PMC4157575

[B39] Ricker B. Mitra S. Castellanos E. A. Grady C. J. Woldring D. Pelled G.. (2023). Proposed three-phenylalanine motif involved in magnetoreception signalling of an Actinopterygii protein expressed in mammalian cells. Open Biol. 13:230019. 10.1098/rsob.230019 37989224 PMC10688439

[B40] Riddle J. Scimeca J. M. Pagnotta M. F. Inglis B. Sheltraw D. Muse-Fisher C.. (2022). A guide for concurrent TMS-fMRI to investigate functional brain networks. Front. Hum. Neurosci. 16:1050605. 10.3389/fnhum.2022.1050605 36590069 PMC9799237

[B41] Samaranch L. Blits B. Sebastian W. S. Hadaczek P. Bringas J. Sudhakar V.. (2017). MR-guided parenchymal delivery of adeno-associated viral vector serotype 5 in non-human primate brain. Gene Ther. 24, 253–261. 10.1038/gt.2017.14 28300083 PMC5404203

[B42] Smith E. J. Farshim P. P. Flomen R. Jones S. T. McAteer S. J. Deverman B. E.. (2021). Use of high-content imaging to quantify transduction of AAV-PHP viruses in the brain following systemic delivery. Brain Commun. 3:fcab105. 10.1093/braincomms/fcab105 34131644 PMC8200048

[B43] Stanley S. A. Sauer J. Kane R. S. Dordick J. S. Friedman J. M. (2015). Corrigendum: Remote regulation of glucose homeostasis in mice using genetically encoded nanoparticles. Nat. Med. 21:537. 10.1038/nm0515-537b 25501906 PMC4894538

[B44] Verley D. R. Torolira D. Pulido B. Gutman B. Bragin A. Mayer A.. (2018). Remote changes in cortical excitability after experimental traumatic brain injury and functional reorganization. J. Neurotrauma 35, 2448–2461. 10.1089/neu.2017.5536 29717625 PMC6196752

[B45] Wheeler M. A. Smith C. J. Ottolini M. Barker B. S. Purohit A. M. Grippo R. M.. (2016). Genetically targeted magnetic control of the nervous system. Nat. Neurosci. 19, 756–761. 10.1038/nn.4265 26950006 PMC4846560

[B46] Zhang X. Pan W. J. Keilholz S. D. (2020). The relationship between BOLD and neural activity arises from temporally sparse events. Neuroimage 207:116390. 10.1016/j.neuroimage.2019.116390 31785420 PMC7252681

[B47] Zhong M. Cywiak C. Metto A. C. Liu X. Qian C. Pelled G.. (2021). Multi-session delivery of synchronous rTMS and sensory stimulation induces long-term plasticity. Brain Stimul. 14, 884–894. 10.1016/j.brs.2021.05.005 34029768 PMC8316373

