# Supplementary material for: Magnetogenetic stimulation inside MRI induces spontaneous and evoked changes in neural circuits activity in rats
Source: Front Neurosci. 2024 Oct 1;18:1459120. doi: 10.3389/fnins.2024.1459120 (PMC11473493; doi:10.3389/fnins.2024.1459120)
Supplement: Supplementary file 1 [file Data_Sheet_1.pdf]

SUPPORTING INFORMATION

Magnetogenetic stimulation inside MRI induces spontaneous and evoked changes in neural circuits activity in rats

Kai-Hsiang Chuang 1,2,3, Chunqi Qian 4, Assaf Gilad 4,5, Galit Pelled 4,6,

[1]Queensland Brain Institute, The University of Queensland, Brisbane, QLD, Australia. kaichuang@gmail.com.

[2]School of Biomedical Sciences, The University of Queensland, Brisbane, QLD, Australia. kaichuang@gmail.com.

[2]Australian Research Council Training Centre for Innovation in Biomedical Imaging Technology, Brisbane, QLD, Australia.

[4]Department of Radiology, Michigan State University, East Lansing, MI, United States

[5]Department of Chemical Engineering and Materials Science, Michigan State University, East Lansing, MI, United States

[6]Department of Mechanical Engineering, Michigan State University, East Lansing, MI, United States

pAAV-CaMKIIα::(EPG(Rat)X3Flag)-IRES-EGFP

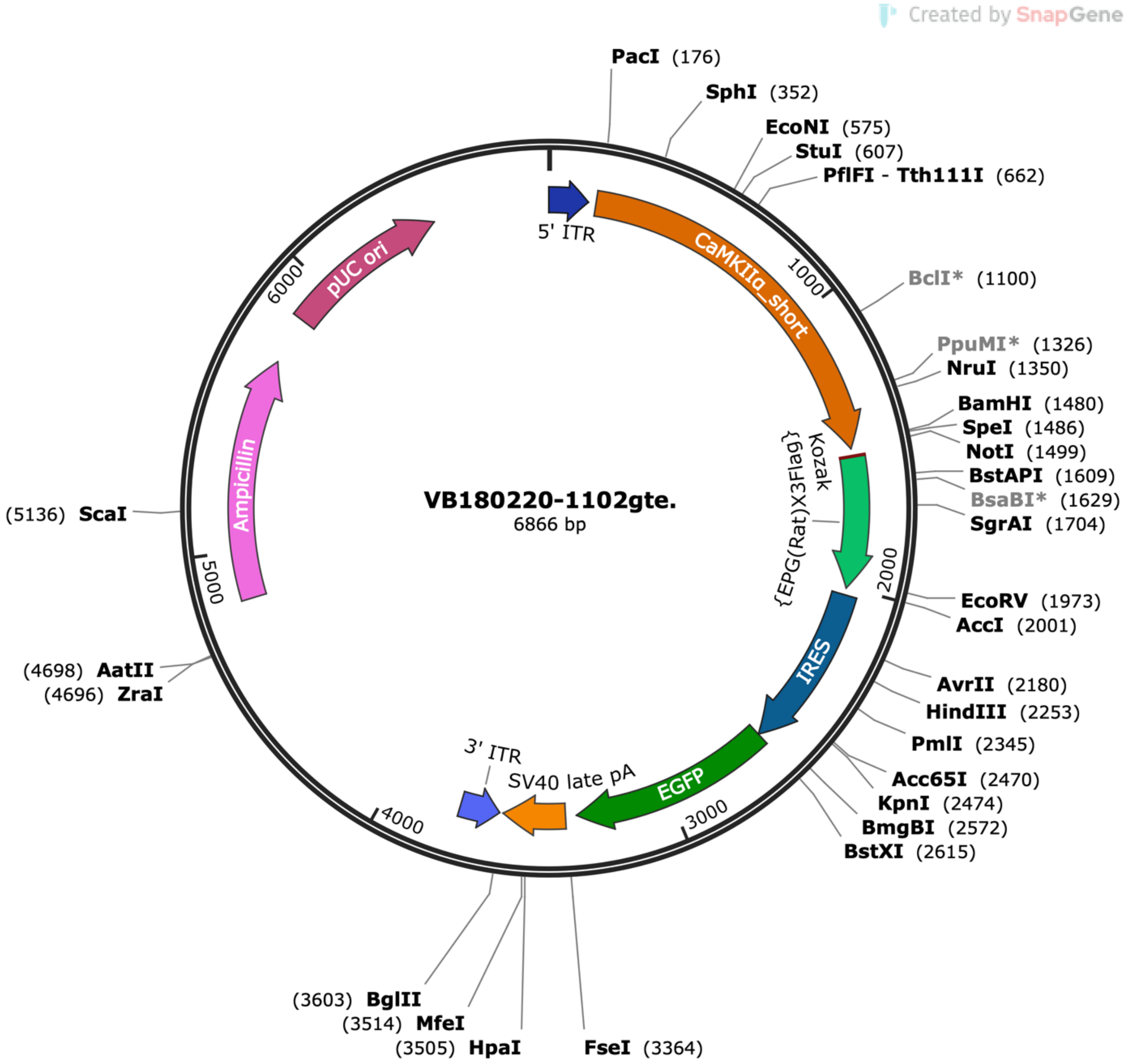

CCTGCAGGCAGCTGCGCGCTCGCTCGCTCACTGAGGCCGCCCGGGCAAAGCCCGGGCGTCGGGCGACCT
TTGGTCGCCCGGCCTCAGTGAGCGAGCGAGCGCGCAGAGAGGGAGTGGCCAACTCCATCACTAGGGGTT
CCTATCGATCAACTTTGTATAGAAAAGTTGCCCTTAATTAACATTATGGCCTTAGGTCACTTCATCTCCATGG
GGTTCTTCTTCTGATTTTCTAGAAAATGAGATGGGGGTGCAGAGAGCTTCCTCAGTGACCTGCCCAGGGT
CACATCAGAAATGTCAGAGCTAGAACTTGAACTCAGATTACTAATCTTAAATTCCATGCCTTGGGGGCATGC
AAGTACGATATACAGAAGGAGTGAACTCATTAGGGCAGATGACCAATGAGTTTAGGAAAGAAGAGTCCAG
GGCAGGGTACATCTACACCACCCGCCCAGCCCTGGGTGAGTCCAGCCACGTTCACCTCATTATAGTTGCCT
CTCTCCAGTCCTACCTTGACGGGAAGCACAAGCAGAAACTGGGACAGGAGCCCCAGGAGACCAAATCTT
CATGGTCCCTCTGGGAGGATGGGTGGGGAGAGCTGTGGCAGAGGCCTCAGGAGGGGCCCTGCTGCTCA
GTGGTGACAGATAGGGGTGAGAAAGCAGACAGAGTCATTCCGTCAGCATTCTGGGTCTGTTTGGTACTTC
TTCTCACGCTAAGGTGGCGGTGTGATATGCACAATGGCTAAAAAGCAGGGAGAGCTGGAAAGAAACAAG

GACAGAGACAGAGGCCAAGTCAACCAGACCAATTCCCAGAGGAAGCAAAGAAACCATTACAGAGACTAC
AAGGGGGAAGGGAAGGAGAGATGAATTAGCTTCCCCTGTAAACCTTAGAACCCAGCTGTTGCCAGGGCA
ACGGGGCAATACCTGTCTCTTCAGAGGAGATGAAGTTGCCAGGGTAACTACATCCTGTCTTTCTCAAGGAC
CATCCCAGAATGTGGCACCCACTAGCCGTTACCATAGCAACTGCCTCTTTGCCCCACTTAATCCCATCCCGTC
TGTTAAAAGGGCCCTATAGTTGGAGGTGGGGGAGGTAGGAAGAGCGATGATCACTTGTGGACTAAGTTT
GTTCGCATCCCCTTCTCCAACCCCCTCAGTACATCACCCTGGGGGAACAGGGTCCACTTGCTCCTGGGCCC
ACACAGTCCTGCAGTATTGTGTATATAAGGCCAGGGCAAAGAGGAGCAGGTTTTAAAGTGAAAGGCAGG
CAGGTGTTGGGGAGGCAGTTACCGGGGCAACGGGAACAGGGCGTTTCGGAGGTGGTTGCCATGGGGAC
CTGGATGCTGACGAAGGCTCGCGAGGCTGTGAGCAGCCACAGTGCCCTGCTCAGAAGCCCCAAGCTCGT
CAGTCAAGCCGGTTCTCCGTTTGCACTCAGGAGCACGGGCAGGCGAGTGGCCCCTAGTTCTGGGGGCAG
CTCTAGAGCGGGGGATCCACTAGTTCTAGAGCGGCCGCCAAGTTTGTACAAAAAAGCAGGCTGCCACCAT
GAAGTGCGTGCTGCTGGGCTTCGCCGCCGTGATCGGCTTCTTCGCCATCGCCGAGAGCCTGACCTGCAAC
ACCTGCAGCGTGAGCCTGATCGGCATCTGCCTGAACCCCGCCACCGCCACCTGCAGCACCAACACCAGCG
TGTGCACCACCGGCAGGGCCAGCTTCACCGGCGTGCTGGGCTTCCTGGGCTTCAACAGCCAGGGCTGCA
CCGAGGGCGCCCAGTGCAACGGCACCGTGAGCGGCAGCATCCTGGGCGCCAGCTACACCGTGACCCAGA
CCTGCTGCAGCACCAACAACTGCAACCCCGTGACCAGCGGCGCCAGCTACGTGCAGATCAGCGTGAGCG
CCGCCCTGAGCGCCGCCCTGCTGGCCTGCGTGTGGGGCCAGAGCGTGTACGACTACAAAGACCATGACG
GTGATTATAAAGATCATGATATCGATTACAAGGATGACGATGACAAGTAGACCCAGCTTTCTTGTACAAAGT
GGGCCCCTCTCCCTCCCCCCCCCCTAACGTTACTGGCCGAAGCCGCTTGGAATAAGGCCGGTGTGCGTTTG
TCTATATGTTATTTTCCACCATATTGCCGTCTTTTGGCAATGTGAGGGCCCGGAAACCTGGCCCTGTCTTCTT
GACGAGCATTCCTAGGGGTCTTTCCCCTCTCGCCAAAGGAATGCAAGGTCTGTTGAATGTCGTGAAGGAA
GCAGTTCCTCTGGAAGCTTCTTGAAGACAAACAACGTCTGTAGCGACCCTTTGCAGGCAGCGGAACCCCC
CACCTGGCGACAGGTGCCTCTGCGGCCAAAAGCCACGTGTATAAGATACACCTGCAAAGGCGGCACAACC
CCAGTGCCACGTTGTGAGTTGGATAGTTGTGGAAAGAGTCAAATGGCTCTCCTCAAGCGTATTCAACAAG
GGGCTGAAGGATGCCCAGAAGGTACCCCATTGTATGGGATCTGATCTGGGGCCTCGGTGCACATGCTTTAC
ATGTGTTTAGTCGAGGTTAAAAAAACGTCTAGGCCCCCCGAACCACGGGGACGTGGTTTTCCTTTGAAAA
ACACGATGATAATATGGCCACAACCATGGTGAGCAAGGGCGAGGAGCTGTTCACCGGGGTGGTGCCCATC
CTGGTCGAGCTGGACGGCGACGTAAACGGCCACAAGTTCAGCGTGTCCGGCGAGGGCGAGGGCGATGC
CACCTACGGCAAGCTGACCCTGAAGTTCATCTGCACCACCGGCAAGCTGCCCGTGCCCTGGCCCACCCTC
GTGACCACCCTGACCTACGGCGTGCAGTGCTTCAGCCGCTACCCCGACCACATGAAGCAGCACGACTTCT
TCAAGTCCGCCATGCCCGAAGGCTACGTCCAGGAGCGCACCATCTTCTTCAAGGACGACGGCAACTACAA
GACCCGCGCCGAGGTGAAGTTCGAGGGCGACACCCTGGTGAACCGCATCGAGCTGAAGGGCATCGACTT
CAAGGAGGACGGCAACATCCTGGGGCACAAGCTGGAGTACAACTACAACAGCCACAACGTCTATATCATG
GCCGACAAGCAGAAGAACGGCATCAAGGTGAACTTCAAGATCCGCCACAACATCGAGGACGGCAGCGTG
CAGCTCGCCGACCACTACCAGCAGAACACCCCCATCGGCGACGGCCCCGTGCTGCTGCCCGACAACCACT
ACCTGAGCACCCAGTCCGCCCTGAGCAAAGACCCCAACGAGAAGCGCGATCACATGGTCCTGCTGGAGTT
CGTGACCGCCGCCGGGATCACTCTCGGCATGGACGAGCTGTACAAGTAACAACTTTATTATACATAGTTGAT
GGCCGGCCGCTTCGAGCAGACATGATAAGATACATTGATGAGTTTGGACAAACCACAACTAGAATGCAGT
GAAAAAAATGCTTTATTTGTGAAATTTGTGATGCTATTGCTTTATTTGTAACCATTATAAGCTGCAATAAACA
AGTTAACAACAACAATTGCATTCATTTTATGTTTCAGGTTCAGGGGGAGGTGTGGGAGGTTTTTTAAAGCA
AGTAAAACCTCTACAAATGTGGTAATCGATAGATCTAGGAACCCCTAGTGATGGAGTTGGCCACTCCCTCTC
TGCGCGCTCGCTCGCTCACTGAGGCCGGGCGACCAAAGGTCGCCCGACGCCCGGGCTTTGCCCGGGCGG
CCTCAGTGAGCGAGCGAGCGCGCAGCTGCCTGCAGGCAGCTTGGCACTGGCCGTCGTTTTACAACGTCG
TGACTGGGAAAACCCTGGCGTTACCCAACTTAATCGCCTTGCAGCACATCCCCCTTTCGCCAGCTGGCGTA

ATAGCGAAGAGGCCCGCACCGATCGCCCTTCCCAACAGTTGCGCAGCCTGAATGGCGAATGGCGCCTGAT
GCGGTATTTTCTCCTTACGCATCTGTGCGGTATTTCACACCGCATACGTCAAAGCAACCATAGTACGCGCCC
TGTAGCGGCGCATTAAGCGCGGCGGGTGTGGTGGTTACGCGCAGCGTGACCGCTACACTTGCCAGCGCC
CTAGCGCCCGCTCCTTTCGCTTTCTTCCCTTCCTTTCTCGCCACGTTCGCCGGCTTTCCCCGTCAAGCTCTAA
ATCGGGGGCTCCCTTTAGGGTTCCGATTTAGTGCTTTACGGCACCTCGACCCCAAAAAACTTGATTTGGGT
GATGGTTCACGTAGTGGGCCATCGCCCTGATAGACGGTTTTTCGCCCTTTGACGTTGGAGTCCACGTTCTT
TAATAGTGGACTCTTGTTCCAAACTGGAACAACACTCAACCCTATCTCGGGCTATTCTTTTGATTTATAAGGG
ATTTTGCCGATTTCGGCCTATTGGTTAAAAAATGAGCTGATTTAACAAAAATTTAACGCGAATTTTAACAAA
ATATTAACGTTTACAATTTTATGGTGCACTCTCAGTACAATCTGCTCTGATGCCGCATAGTTAAGCCAGCCCC
GACACCCGCCAACACCCGCTGACGCGCCCTGACGGGCTTGTCTGCTCCCGGCATCCGCTTACAGACAAGC
TGTGACCGTCTCCGGGAGCTGCATGTGTCAGAGGTTTTCACCGTCATCACCGAAACGCGCGAGACGAAAG
GGCCTCGTGATACGCCTATTTTTATAGGTTAATGTCATGATAATAATGGTTTCTTAGACGTCAGGTGGCACTT
TTCGGGGAAATGTGCGCGGAACCCCTATTTGTTTATTTTTCTAAATACATTCAAATATGTATCCGCTCATGAG
ACAATAACCCTGATAAATGCTTCAATAATATTGAAAAAGGAAGAGTATGAGTATTCAACATTTCCGTGTCGC
CCTTATTCCCTTTTTTGCGGCATTTTGCCTTCCTGTTTTTGCTCACCCAGAAACGCTGGTGAAAGTAAAAGA
TGCTGAAGATCAGTTGGGTGCACGAGTGGGTTACATCGAACTGGATCTCAACAGCGGTAAGATCCTTGAG
AGTTTTCGCCCCGAAGAACGTTTTCCAATGATGAGCACTTTTAAAGTTCTGCTATGTGGCGCGGTATTATCC
CGTATTGACGCCGGGCAAGAGCAACTCGGTCGCCGCATACACTATTCTCAGAATGACTTGGTTGAGTACTC
ACCAGTCACAGAAAAGCATCTTACGGATGGCATGACAGTAAGAGAATTATGCAGTGCTGCCATAACCATGA
GTGATAACACTGCGGCCAACTTACTTCTGACAACGATCGGAGGACCGAAGGAGCTAACCGCTTTTTTGCA
CAACATGGGGGATCATGTAACTCGCCTTGATCGTTGGGAACCGGAGCTGAATGAAGCCATACCAAACGAC
GAGCGTGACACCACGATGCCTGTAGCAATGGCAACAACGTTGCGCAAACTATTAACTGGCGAACTACTTAC
TCTAGCTTCCCGGCAACAATTAATAGACTGGATGGAGGCGGATAAAGTTGCAGGACCACTTCTGCGCTCG
GCCCTTCCGGCTGGCTGGTTTATTGCTGATAAATCTGGAGCCGGTGAGCGTGGGTCTCGCGGTATCATTGC
AGCACTGGGGCCAGATGGTAAGCCCTCCCGTATCGTAGTTATCTACACGACGGGGAGTCAGGCAACTATG
GATGAACGAAATAGACAGATCGCTGAGATAGGTGCCTCACTGATTAAGCATTGGTAACTGTCAGACCAAGT
TTACTCATATATACTTTAGATTGATTTAAAACTTCATTTTTAATTTAAAAGGATCTAGGTGAAGATCCTTTTTG
ATAATCTCATGACCAAAATCCCTTAACGTGAGTTTTCGTTCCACTGAGCGTCAGACCCCGTAGAAAAGATCA
AAGGATCTTCTTGAGATCCTTTTTTTCTGCGCGTAATCTGCTGCTTGCAAACAAAAAAACCACCGCTACCAG
CGGTGGTTTGTTTGCCGGATCAAGAGCTACCAACTCTTTTTCCGAAGGTAACTGGCTTCAGCAGAGCGCA
GATACCAAATACTGTTCTTCTAGTGTAGCCGTAGTTAGGCCACCACTTCAAGAACTCTGTAGCACCGCCTAC
ATACCTCGCTCTGCTAATCCTGTTACCAGTGGCTGCTGCCAGTGGCGATAAGTCGTGTCTTACCGGGTTGGA
CTCAAGACGATAGTTACCGGATAAGGCGCAGCGGTCGGGCTGAACGGGGGGTTCGTGCACACAGCCCAG
CTTGGAGCGAACGACCTACACCGAACTGAGATACCTACAGCGTGAGCTATGAGAAAGCGCCACGCTTCCC
GAAGGGAGAAAGGCGGACAGGTATCCGGTAAGCGGCAGGGTCGGAACAGGAGAGCGCACGAGGGAG
CTTCCAGGGGGAAACGCCTGGTATCTTTATAGTCCTGTCGGGTTTCGCCACCTCTGACTTGAGCGTCGATTT
TTGTGATGCTCGTCAGGGGGGCGGAGCCTATGGAAAAACGCCAGCAACGCGGCCTTTTTACGGTTCCTGG
CCTTTTGCTGGCCTTTTGCTCACATGTTCTTTCCTGCGTTATCCCCTGATTCTGTGGATAACCGTATTACCGCC
TTTGAGTGAGCTGATACCGCTCGCCGCAGCCGAACGACCGAGCGCAGCGAGTCAGTGAGCGAGGAAGC
GGAAGAGCGCCCAATACGCAAACCGCCTCTCCCCGCGCGTTGGCCGATTCATTAATGCAGCTGGCACGAC
AGGTTTCCCGACTGGAAAGCGGGCAGTGAGCGCAACGCAATTAATGTGAGTTAGCTCACTCATTAGGCAC
CCCAGGCTTTACACTTTATGCTTCCGGCTCGTATGTTGTGTGGAATTGTGAGCGGATAACAATTTCACACAG
GAAACAGCTATGACCATGATTACGAATTG

pAAV-CaMKIIα::EGFP

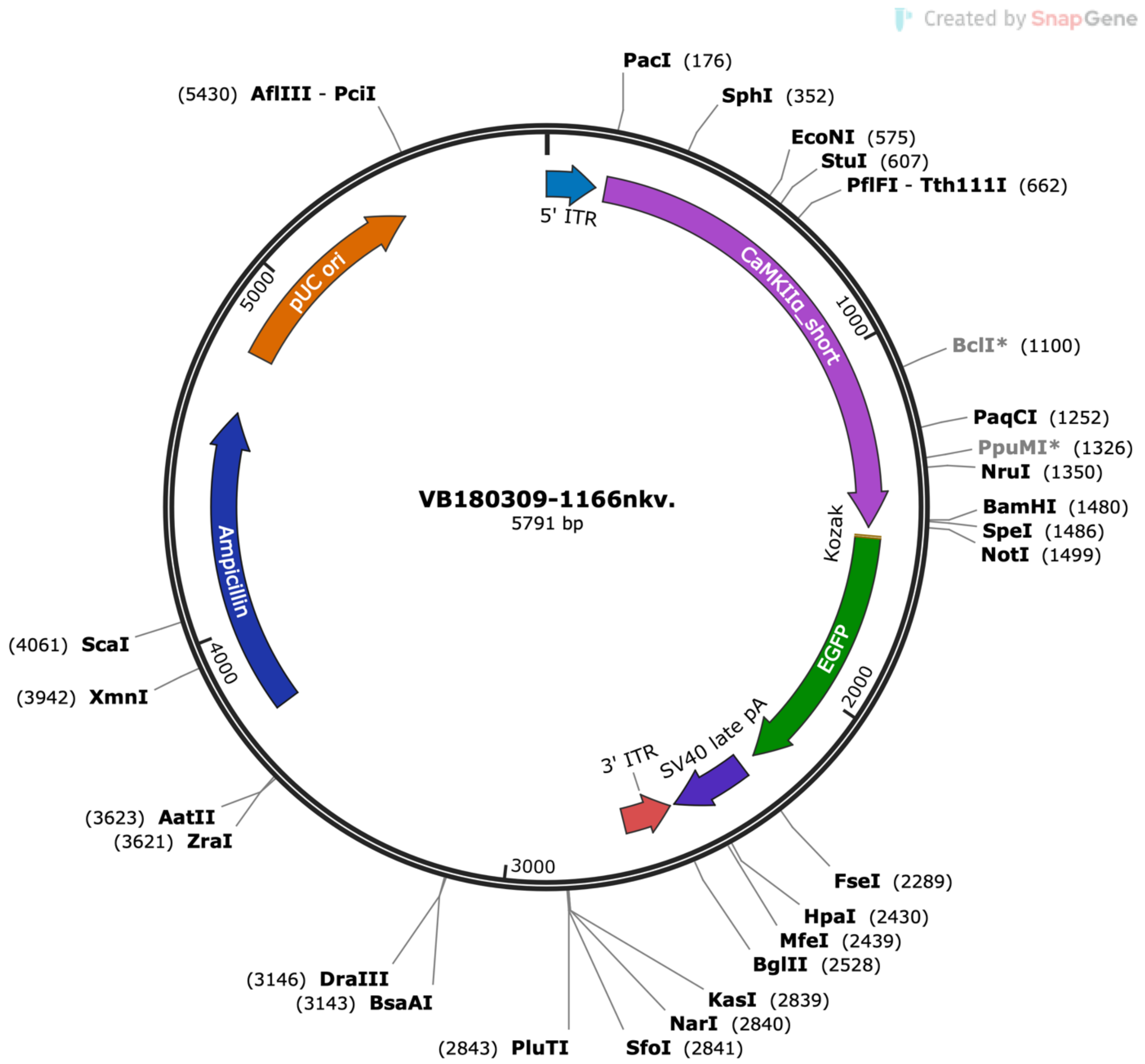

CCTGCAGGCAGCTGCGCGCTCGCTCGCTCACTGAGGCCGCCCGGGCAAAGCCCGGGCGTCGGGCGACCT
TTGGTCGCCCGGCCTCAGTGAGCGAGCGAGCGCGCAGAGAGGGAGTGGCCAACTCCATCACTAGGGGTT
CCTATCGATCAACTTTGTATAGAAAAGTTGCCCTTAATTAACATTATGGCCTTAGGTCACTTCATCTCCATGG
GGTTCTTCTTCTGATTTTCTAGAAAATGAGATGGGGGTGCAGAGAGCTTCCTCAGTGACCTGCCCAGGGT
CACATCAGAAATGTCAGAGCTAGAACTTGAACTCAGATTACTAATCTTAAATTCCATGCCTTGGGGGCATGC
AAGTACGATATACAGAAGGAGTGAACTCATTAGGGCAGATGACCAATGAGTTTAGGAAAGAAGAGTCCAG
GGCAGGGTACATCTACACCACCCGCCCAGCCCTGGGTGAGTCCAGCCACGTTCACCTCATTATAGTTGCCT
CTCTCCAGTCCTACCTTGACGGGAAGCACAAGCAGAAACTGGGACAGGAGCCCCAGGAGACCAAATCTT
CATGGTCCCTCTGGGAGGATGGGTGGGGAGAGCTGTGGCAGAGGCCTCAGGAGGGGCCCTGCTGCTCA
GTGGTGACAGATAGGGGTGAGAAAGCAGACAGAGTCATTCCGTCAGCATTCTGGGTCTGTTTGGTACTTC
TTCTCACGCTAAGGTGGCGGTGTGATATGCACAATGGCTAAAAAGCAGGGAGAGCTGGAAAGAAACAAG
GACAGAGACAGAGGCCAAGTCAACCAGACCAATTCCCAGAGGAAGCAAAGAAACCATTACAGAGACTAC

AAGGGGGAAGGGAAGGAGAGATGAATTAGCTTCCCCTGTAAACCTTAGAACCCAGCTGTTGCCAGGGCA
ACGGGGCAATACCTGTCTCTTCAGAGGAGATGAAGTTGCCAGGGTAACTACATCCTGTCTTTCTCAAGGAC
CATCCCAGAATGTGGCACCCACTAGCCGTTACCATAGCAACTGCCTCTTTGCCCCACTTAATCCCATCCCGTC
TGTTAAAAGGGCCCTATAGTTGGAGGTGGGGGAGGTAGGAAGAGCGATGATCACTTGTGGACTAAGTTT
GTTCGCATCCCCTTCTCCAACCCCCTCAGTACATCACCCTGGGGGAACAGGGTCCACTTGCTCCTGGGCCC
ACACAGTCCTGCAGTATTGTGTATATAAGGCCAGGGCAAAGAGGAGCAGGTTTTAAAGTGAAAGGCAGG
CAGGTGTTGGGGAGGCAGTTACCGGGGCAACGGGAACAGGGCGTTTCGGAGGTGGTTGCCATGGGGAC
CTGGATGCTGACGAAGGCTCGCGAGGCTGTGAGCAGCCACAGTGCCCTGCTCAGAAGCCCCAAGCTCGT
CAGTCAAGCCGGTTCTCCGTTTGCACTCAGGAGCACGGGCAGGCGAGTGGCCCCTAGTTCTGGGGGCAG
CTCTAGAGCGGGGGATCCACTAGTTCTAGAGCGGCCGCCAAGTTTGTACAAAAAAGCAGGCTGCCACCAT
GGTGAGCAAGGGCGAGGAGCTGTTCACCGGGGTGGTGCCCATCCTGGTCGAGCTGGACGGCGACGTAA
ACGGCCACAAGTTCAGCGTGTCCGGCGAGGGCGAGGGCGATGCCACCTACGGCAAGCTGACCCTGAAGT
TCATCTGCACCACCGGCAAGCTGCCCGTGCCCTGGCCCACCCTCGTGACCACCCTGACCTACGGCGTGCAG
TGCTTCAGCCGCTACCCCGACCACATGAAGCAGCACGACTTCTTCAAGTCCGCCATGCCCGAAGGCTACGT
CCAGGAGCGCACCATCTTCTTCAAGGACGACGGCAACTACAAGACCCGCGCCGAGGTGAAGTTCGAGGG
CGACACCCTGGTGAACCGCATCGAGCTGAAGGGCATCGACTTCAAGGAGGACGGCAACATCCTGGGGCA
CAAGCTGGAGTACAACTACAACAGCCACAACGTCTATATCATGGCCGACAAGCAGAAGAACGGCATCAAG
GTGAACTTCAAGATCCGCCACAACATCGAGGACGGCAGCGTGCAGCTCGCCGACCACTACCAGCAGAAC
ACCCCCATCGGCGACGGCCCCGTGCTGCTGCCCGACAACCACTACCTGAGCACCCAGTCCGCCCTGAGCA
AAGACCCCAACGAGAAGCGCGATCACATGGTCCTGCTGGAGTTCGTGACCGCCGCCGGGATCACTCTCGG
CATGGACGAGCTGTACAAGTAAACCCAGCTTTCTTGTACAAAGTGGTGATGGCCGGCCGCTTCGAGCAGA
CATGATAAGATACATTGATGAGTTTGGACAAACCACAACTAGAATGCAGTGAAAAAAATGCTTTATTTGTGA
AATTTGTGATGCTATTGCTTTATTTGTAACCATTATAAGCTGCAATAAACAAGTTAACAACAACAATTGCATTC
ATTTTATGTTTCAGGTTCAGGGGGAGGTGTGGGAGGTTTTTTAAAGCAAGTAAAACCTCTACAAATGTGGT
AATCGATAGATCTAGGAACCCCTAGTGATGGAGTTGGCCACTCCCTCTCTGCGCGCTCGCTCGCTCACTGA
GGCCGGGCGACCAAAGGTCGCCCGACGCCCGGGCTTTGCCCGGGCGGCCTCAGTGAGCGAGCGAGCGC
GCAGCTGCCTGCAGGCAGCTTGGCACTGGCCGTCGTTTTACAACGTCGTGACTGGGAAAACCCTGGCGTT
ACCCAACTTAATCGCCTTGCAGCACATCCCCCTTTCGCCAGCTGGCGTAATAGCGAAGAGGCCCGCACCGA
TCGCCCTTCCCAACAGTTGCGCAGCCTGAATGGCGAATGGCGCCTGATGCGGTATTTTCTCCTTACGCATCT
GTGCGGTATTTCACACCGCATACGTCAAAGCAACCATAGTACGCGCCCTGTAGCGGCGCATTAAGCGCGGC
GGGTGTGGTGGTTACGCGCAGCGTGACCGCTACACTTGCCAGCGCCCTAGCGCCCGCTCCTTTCGCTTTCT
TCCCTTCCTTTCTCGCCACGTTCGCCGGCTTTCCCCGTCAAGCTCTAAATCGGGGGCTCCCTTTAGGGTTCC
GATTTAGTGCTTTACGGCACCTCGACCCCAAAAAACTTGATTTGGGTGATGGTTCACGTAGTGGGCCATCG
CCCTGATAGACGGTTTTTCGCCCTTTGACGTTGGAGTCCACGTTCTTTAATAGTGGACTCTTGTTCCAAACT
GGAACAACACTCAACCCTATCTCGGGCTATTCTTTTGATTTATAAGGGATTTTGCCGATTTCGGCCTATTGGT
TAAAAAATGAGCTGATTTAACAAAAATTTAACGCGAATTTTAACAAAATATTAACGTTTACAATTTTATGGTG
CACTCTCAGTACAATCTGCTCTGATGCCGCATAGTTAAGCCAGCCCCGACACCCGCCAACACCCGCTGACG
CGCCCTGACGGGCTTGTCTGCTCCCGGCATCCGCTTACAGACAAGCTGTGACCGTCTCCGGGAGCTGCAT
GTGTCAGAGGTTTTCACCGTCATCACCGAAACGCGCGAGACGAAAGGGCCTCGTGATACGCCTATTTTTAT
AGGTTAATGTCATGATAATAATGGTTTCTTAGACGTCAGGTGGCACTTTTCGGGGAAATGTGCGCGGAACC
CCTATTTGTTTATTTTTCTAAATACATTCAAATATGTATCCGCTCATGAGACAATAACCCTGATAAATGCTTCAA
TAATATTGAAAAAGGAAGAGTATGAGTATTCAACATTTCCGTGTCGCCCTTATTCCCTTTTTTGCGGCATTTT
GCCTTCCTGTTTTTGCTCACCCAGAAACGCTGGTGAAAGTAAAAGATGCTGAAGATCAGTTGGGTGCACG
AGTGGGTTACATCGAACTGGATCTCAACAGCGGTAAGATCCTTGAGAGTTTTCGCCCCGAAGAACGTTTTC

CAATGATGAGCACTTTTAAAGTTCTGCTATGTGGCGCGGTATTATCCCGTATTGACGCCGGGCAAGAGCAA
CTCGGTCGCCGCATACACTATTCTCAGAATGACTTGGTTGAGTACTCACCAGTCACAGAAAAGCATCTTACG
GATGGCATGACAGTAAGAGAATTATGCAGTGCTGCCATAACCATGAGTGATAACACTGCGGCCAACTTACT
TCTGACAACGATCGGAGGACCGAAGGAGCTAACCGCTTTTTTGCACAACATGGGGGATCATGTAACTCGC
CTTGATCGTTGGGAACCGGAGCTGAATGAAGCCATACCAAACGACGAGCGTGACACCACGATGCCTGTAG
CAATGGCAACAACGTTGCGCAAACTATTAACTGGCGAACTACTTACTCTAGCTTCCCGGCAACAATTAATAG
ACTGGATGGAGGCGGATAAAGTTGCAGGACCACTTCTGCGCTCGGCCCTTCCGGCTGGCTGGTTTATTGC
TGATAAATCTGGAGCCGGTGAGCGTGGGTCTCGCGGTATCATTGCAGCACTGGGGCCAGATGGTAAGCCC
TCCCGTATCGTAGTTATCTACACGACGGGGAGTCAGGCAACTATGGATGAACGAAATAGACAGATCGCTGA
GATAGGTGCCTCACTGATTAAGCATTGGTAACTGTCAGACCAAGTTTACTCATATATACTTTAGATTGATTTA
AAACTTCATTTTTAATTTAAAAGGATCTAGGTGAAGATCCTTTTTGATAATCTCATGACCAAAATCCCTTAAC
GTGAGTTTTCGTTCCACTGAGCGTCAGACCCCGTAGAAAAGATCAAAGGATCTTCTTGAGATCCTTTTTTT
CTGCGCGTAATCTGCTGCTTGCAAACAAAAAAACCACCGCTACCAGCGGTGGTTTGTTTGCCGGATCAAG
AGCTACCAACTCTTTTTCCGAAGGTAACTGGCTTCAGCAGAGCGCAGATACCAAATACTGTTCTTCTAGTGT
AGCCGTAGTTAGGCCACCACTTCAAGAACTCTGTAGCACCGCCTACATACCTCGCTCTGCTAATCCTGTTAC
CAGTGGCTGCTGCCAGTGGCGATAAGTCGTGTCTTACCGGGTTGGACTCAAGACGATAGTTACCGGATAA
GGCGCAGCGGTCGGGCTGAACGGGGGGTTCGTGCACACAGCCCAGCTTGGAGCGAACGACCTACACCG
AACTGAGATACCTACAGCGTGAGCTATGAGAAAGCGCCACGCTTCCCGAAGGGAGAAAGGCGGACAGGT
ATCCGGTAAGCGGCAGGGTCGGAACAGGAGAGCGCACGAGGGAGCTTCCAGGGGGAAACGCCTGGTAT
CTTTATAGTCCTGTCGGGTTTCGCCACCTCTGACTTGAGCGTCGATTTTTGTGATGCTCGTCAGGGGGGCG
GAGCCTATGGAAAAACGCCAGCAACGCGGCCTTTTTACGGTTCCTGGCCTTTTGCTGGCCTTTTGCTCACA
TGTTCTTTCCTGCGTTATCCCCTGATTCTGTGGATAACCGTATTACCGCCTTTGAGTGAGCTGATACCGCTCG
CCGCAGCCGAACGACCGAGCGCAGCGAGTCAGTGAGCGAGGAAGCGGAAGAGCGCCCAATACGCAAAC
CGCCTCTCCCCGCGCGTTGGCCGATTCATTAATGCAGCTGGCACGACAGGTTTCCCGACTGGAAAGCGGG
CAGTGAGCGCAACGCAATTAATGTGAGTTAGCTCACTCATTAGGCACCCCAGGCTTTACACTTTATGCTTCC
GGCTCGTATGTTGTGTGGAATTGTGAGCGGATAACAATTTCACACAGGAAACAGCTATGACCATGATTACG
AATTG

Supplementary Table S1
The full list of gray matter regions in each hemisphere used in the ROI analysis.

ROI	Abbreviation
Agranular Dysgranular Insular Cortex	ADI
Agranular Insular Cortex	AI
Amygdalohyppocampic Area	AHi
Amygdalopiriform Cortex	APir
Basal Forebrain Region	BF
Bed Nucleus of the Stria Terminalis	BNST
Cornu Ammonis 1	CA1
Cornu Ammonis 2	CA2
Cornu Ammonis 3	CA3
Dentate Gyrus	DG
Dorso Lateral Orbital Cortex	LO
Dysgranular Insular Cortex	DI
Ectorhinal Cortex	Ect
Entorhinal Cortex	Ent
Fasciola Cinereum	FaC
Frontal Association Cortex	FrA
Globus Pallidus	GP
Glomerular Layer of the Accessory Olfactory Bulb	AOB
Glomerular Layer of the Olfactory Bulb	OB
Lateral Temporal Associative Cortex	TeAL
Lateral Primary Auditory Cortex	Au1
Granule Cell Level of the Cerebellum	CeG
Hypothalamic Region	Hy
Interpeduncular Nucleus	IPN
Lateral Entorhinal Cortex Internal part	LEntIn
Lateral Entorhinal Cortex	LEnt
Lateral Entorhinal Cortex external part	LEntEx
Lateral Parietal Associative Cortex	LPtA
Lateral Secondary Visual Cortex	V2L
Medial Entorhinal Cortex	Ment
Medial Parietal Associative Cortex	MPtA
Medio Lateral Secondary Visual Cortex	V2ML
Medio Medial Secondary Visual Cortex	V2MM
Molecular Cell Level of the Cerebellum	CeM
Olfactory Bulb	OB
Orbitofrontal Region	OFC
Parasubiculum	PaS
Parietal Cortex Postero Caudal Part	PtPC
Parietal Cortex Postero Dorsal Part	PtPD
Parietal Cortex Postero Rostral	PtPR

Periaqueductal Gray	PAG
Perirhinal Area 35	PRh35
Perirhinal Area 36	PRh36
Perirhinal Cortex	PRh
Posterior Agralunar Insular Cortex	AIP
PreLimbic System	PrL
Presubiculum	PrS
Pretectal Region	PrT
Primary Auditory Cortex	AUD
Primary Cingular Cortex	Cg1
Primary Motor Cortex	M1
Primary Somatosensory Cortex Barrel field	S1BF
Primary Somatosensory Cortex Dysgranular	S1DZ
Primary Somatosensory Cortex Dysgranular Zone 0	S1DZ0
Primary Somatosensory Cortex Forelimb	S1FL
Primary Somatosensory Cortex Hindlimb	S1HL
Primary Somatosensory Cortex Jaw	S1J
Primary Somatosensory Cortex	S1C
Primary Somatosensory Cortex Shoulder	S1Sh
Primary Somatosensory Cortex Trunk	S1Tr
Primary Somatosensory Cortex Upperlips	S1ULp
Primary Visual Cortex Binocular Area	V1b
Primary Visual Cortex	V1c
Primary Visual Cortex Monocular Area	V1m
Retosplenial Dysgranular Cortex	RSCd
Retrosplenial Granular Cortex Part A	RSCa
Retrosplenial Granular Cortex Part B	RSCb
Secondary Auditory Cortex Dorsal Part	Au2D
Secondary Auditory Cortex Ventral Part	Au2V
Secondary Cingular Cortex	Cg2
Secondary Motor Cortex	M2
Secondary Somatosensory Cortex	S2
Striatum	STR
Subiculum	Sub
Substantia Nigra	SN
Superficial Gray Layer of the Superior Colliculus	SCs
Temporal Associative Cortex	TeA
Brainstem	BS
Deeper Layers of the Superior Colliculus	SCd
External Cortex of the Inferior Colliculus	ICe
Septal Region	Sep
Subthalamic Nucleus	STN
Thalamus	TH

Periventricular Grey	PVG
Pons	Pons
